# Lymphopenia predicts disease severity of COVID-19: a descriptive and predictive study

**DOI:** 10.1038/s41392-020-0148-4

**Published:** 2020-03-27

**Authors:** Li Tan, Qi Wang, Duanyang Zhang, Jinya Ding, Qianchuan Huang, Yi-Quan Tang, Qiongshu Wang, Hongming Miao

**Affiliations:** 1 Department of Disease Control and Prevention, General Hospital of Central Theater Command, 430015 Wuhan, Hubei province People’s Republic of China; 2 Department of Laboratory, General Hospital of Central Theater Command, 430015 Wuhan, Hubei province People’s Republic of China; 3 MRC Laboratory of Molecular Biology, Cambridge Biomedical Campus, Cambridge, CB2 0QH UK; 4 Department of Biochemistry and Molecular Biology, Third Military Medical University (Army Medical University), 400038 Chongqing, People’s Republic of China

**Keywords:** Infectious diseases, Immunological disorders

**Dear Editor**,

An outbreak of an unknown infectious pneumonia has recently occurred in Wuhan, China.^1^ The pathogen of the disease was quickly identified as a novel coronavirus (SARS-CoV-2, severe acute respiratory syndrome coronavirus 2), and the disease was named coronavirus disease-19 (COVID-19).^2^ The virus has so far caused 78,959 confirmed cases and 2791 deaths in China according to the reports of government. COVID-19 has been spreading in many countries such as Japan, Korea, Singapore, Iran, and Italia. The clinical manifestation of COVID-19 include fever, cough, fatigue, muscle pain, diarrhea, and pneumonia, which can develop to acute respiratory distress syndrome, metabolic acidosis, septic shock, coagulation dysfunction, and organ failure such as liver, kidney, and heart failure.^1,3,4^ Unfortunately, there is no effective medication other than comprehensive support. However, the mild type of COVID-19 patients can recover shortly after appropriate clinical intervention. The moderate type patients, especially the elderly or the ones with comorbidity, can worsen and became severe, indicating high mortality rate.^3,4^ However, efficient indicators for the disease severity, therapeutic response and disease outcome have not been fully investigated. Once such indicators are present, reasonable medication and care can be inclined, which is believed to significantly reduce the mortality rate of severe patients.

Routine examinations include complete blood count, coagulation profile, and serum biochemical test (including renal and liver function, creatine kinase, lactate dehydrogenase, and electrolytes). Complete blood count is the most available, efficient and economic examination. This study aims to retrospect and analyze the time-courses of complete blood count of cured and dead patients, in order to obtain key indicators of disease progression and outcome and to provide guidance for subsequent clinical practice.

## Low LYM% is a predictor of prognosis in COVID-19 patients

We first randomly selected five death cases and monitored dynamic changes in blood tests for each patient from disease onset to death. Although course of disease in each patient was different, inter-day variations of most parameters studied are fairly constant among all five patients (Supplementary Fig. S1a–f). Among all parameters, blood lymphocyte percentage (LYM%) showed the most significant and consistent trend (Supplementary Fig. S1f), suggesting that this indicator might reflect the disease progression. To further confirm the relationship between blood LYM% and patient’s condition, we increased our sample size to 12 death cases (mean age: 76 years; average therapeutic time: 20 days) (Supplementary Table S1). Most cases showed that LYM% was reduced to lower than 5% within 2 weeks after disease onset (Supplementary Fig. S2a). We also randomly selected seven cases (mean age: 35 years, average therapeutic time: 35 days) with severe symptoms and treatment outcomes (Supplementary Table S2) and 11 cases (mean age: 49; average therapeutic time: 26 days) with moderate symptoms and treatment outcomes (Supplementary Table S3). LYM% of severe patients decreased initially and then increased to higher than 10% until discharged (Supplementary Fig. S2b). In contrast, LYM% of moderate patients fluctuated very little after disease onset and was higher than 20% when discharged (Supplementary Fig. S2c). These results suggest that lymphopenia is a predictor of prognosis in COVID-19 patients.

## Establishment of a Time-LYM% model from discharged COVID-19 patients

**Fig. 1:**
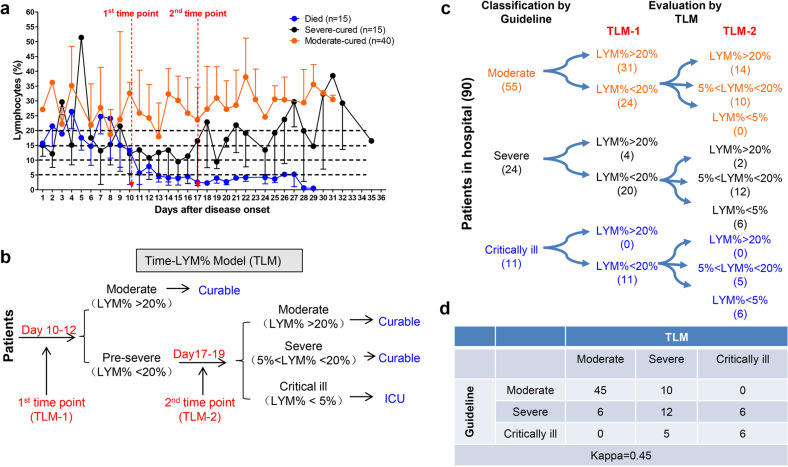
Establishment and validation of Time-LYM% model (TLM) in patients with COVID-19. **a** Dynamic changes of LYM% in the death cases (*n* = 15), severe-cured cases (*n* = 15), and moderate-cured cases (*n* = 40). Data are showen as means ± s.e.ms. Two cutoff time points of these three curves were set as 1st time point (day 10–12) and 2nd time point (day 17–19). **b** Description of TLM: 1st time point (TLM-1) and 2nd time point (TLM-2) are defined as day 10–12 and day 17–19 from symptom onset, respectively. The confirmed COVID-19 patients with LYM% > 20% at TLM-1 are classified as moderate type and the ones with LYM% < 20% at TLM-1 are suggested as pre-severe type, which need to be further distinguished at TLM-2. If LYM% > 20% at TLM-2, those pre-severe patients are reclassified as moderate. If 5% < LYM% < 20% at TLM-2, the pre-severe patients are indeed typed as severe. If LYM% < 5% at TLM-2, those patients are suggested as critically ill. The moderate and severe types are curable, while the critically ill types need intensive care has a poor prognosis. **c** Ninety COVID-19 patients were currently hospitalized in light of the classification criteria of the New Coronavirus Pneumonia Diagnosis Program (5th edition): 55 patients with moderate type, 24 patients with severe type and 11 patients with critically ill type. At TLM-1, LYM% in 24 out of 55 moderate cases was lower than 20%; At TLM-2, LYM% in all 24 patients was above 5%, indicating that these patients would be curable. Regarding other 24 patients with severe symptoms, LYM% at TLM-1 was lower than 20% in 20 out of 24 cases. LYM% at TLM-2 in 6 cases was <5%, indicating a poor prognosis. In 11 out of 11 critically ill patients, LYM% at TLM-1 was lower than 20%. LYM% at TLM-2 in six cases was lower than 5%, suggesting a poor prognosis. **d** The consistency between Guideline and TLM-based disease classification in **c** was tested using kappa statistic. Kappa = 0.48; *P* < 0.005

By summarizing all the death and cured cases in our hospital to depict the time-LYM% curve (Fig. 1a), we established a Time-LYM% model (TLM) for disease classification and prognosis prediction (Fig. 1b). We defined TLM as follows: patients have varying LYM% after the onset of COVID-19. At the 1st time point (TLM-1) of 10–12 days after symptom onset, patients with LYM% > 20% are classified as moderate type and can recover quickly. Patients with LYM% < 20% are initially classified as severe type. At the 2nd time point (TLM-2) of 17–19 days after symptom onset, patients with LYM% > 20% are in recovery; patients with 5% < LYM% < 20% are still in danger and in need of supervision; patients with LYM% < 5% become critically ill with high mortality rate and need intensive care.

## Validation of TLM in disease classification in hospitalized COVID-19 patients

To validate the reliability of TLM, 90 hospitalized COVID-19 patients typed by the latest classification guideline (5th edition) were redefined with TLM. LYM% in 24 out of 55 moderate cases was lower than 20% at TLM-1; LYM% of all these patients was above 5% at TLM-2, indicating that these patients would recover soon. LYM% at TLM-1 was lower than 20% in 20 out of 24 severe cases; LYM% at TLM-2 was <5% in six cases, indicating a poor prognosis. LYM% at TLM-1 in 11 out of 11 critically ill patients was lower than 20%; LYM% of these patients at TLM-2 was lower than 5% in six cases, suggesting a poor outcome (Fig. 1c). Furthermore, with kappa statistic test, we verified the consistency between TLM and the existing guideline in disease typing (Fig. 1d).

## LYM% indicates disease severity of COVID-19 patients

The classification of disease severity in COVID-19 is very important for the grading treatment of patients. In particular, when the outbreak of an epidemic occurs and medical resources are relatively scarce, it is necessary to conduct grading severity and treatment, thereby optimize the allocation of rescue resources and prevent the occurrence of overtreatment or undertreatment. According to the latest 5th edition of the national treatment guideline, COVID-19 can be classified into four types. Pulmonary imaging is the main basis of classification, and other auxiliary examinations are used to distinguish the severity. Blood tests are easy, fast, and cost-effective. However, none of the indicators in blood tests were included in the classification criteria. This study suggested that LYM% can be used as a reliable indicator to classify the moderate, severe, and critical ill types independent of any other auxiliary indicators.

## Analysis of possible reasons for lymphopenia in COVID-19 patients

Lymphocytes play a decisive role in maintaining immune homeostasis and inflammatory response throughout the body. Understanding the mechanism of reduced blood lymphocyte levels is expected to provide an effective strategy for the treatment of COVID-19. We speculated four potential mechanisms leading to lymphocyte deficiency. (1) The virus might directly infect lymphocytes, resulting in lymphocyte death. Lymphocytes express the coronavirus receptor ACE2 and may be a direct target of viruses.^5^ (2) The virus might directly destroy lymphatic organs. Acute lymphocyte decline might be related to lymphocytic dysfunction, and the direct damage of novel coronavirus virus to organs such as thymus and spleen cannot be ruled out. This hypothesis needs to be confirmed by pathological dissection in the future. (3) Inflammatory cytokines continued to be disordered, perhaps leading to lymphocyte apoptosis. Basic researches confirmed that tumour necrosis factor (TNF)α, interleukin (IL)-6, and other pro-inflammatory cytokines could induce lymphocyte deficiency.^6^ (4) Inhibition of lymphocytes by metabolic molecules produced by metabolic disorders, such as hyperlactic acidemia. The severe type of COVID-19 patients had elevated blood lactic acid levels, which might suppress the proliferation of lymphocytes.^7^ Multiple mechanisms mentioned above or beyond might work together to cause lymphopenia, and further research is needed.

In conclusion, lymphopenia is an effective and reliable indicator of the severity and hospitalization in COVID-19 patients. We suggest that TLM should be included in the diagnosis and therapeutic guidelines of COVID-19.

## Supplementary information

Supplementary information

## References

[1] Chen N (2020). Epidemiological and clinical characteristics of 99 cases of 2019 novel coronavirus pneumonia in Wuhan, China: a descriptive study. Lancet.

[2] Zhu N (2020). A novel coronavirus from patients with pneumonia in China, 2019. N. Engl. J. Med..

[3] Wang D (2020). Clinical characteristics of 138 hospitalized patients with 2019 novel coronavirus-infected pneumonia in Wuhan, China. JAMA.

[4] Huang C (2020). Clinical features of patients infected with 2019 novel coronavirus in Wuhan, China. Lancet.

[5] Xu H (2020). High expression of ACE2 receptor of 2019-nCoV on the epithelial cells of oral mucosa. Int J. Oral. Sci..

[6] Liao YC (2002). IL-19 induces production of IL-6 and TNF-alpha and results in cell apoptosis through TNF-alpha. J. Immunol..

[7] Fischer K (2007). Inhibitory effect of tumor cell-derived lactic acid on human T cells. Blood.

