# Correction: Lymphopenia predicts disease severity of COVID-19: a descriptive and predictive study

**DOI:** 10.1038/s41392-020-0159-1

**Published:** 2020-04-29

**Authors:** Li Tan, Qi Wang, Duanyang Zhang, Jinya Ding, Qianchuan Huang, Yi-Quan Tang, Qiongshu Wang, Hongming Miao

**Affiliations:** 1 Department of Disease Control and Prevention, General Hospital of Central Theater Command, 430015 Wuhan, Hubei province People’s Republic of China; 2 Department of Laboratory, General Hospital of Central Theater Command, 430015 Wuhan, Hubei province People’s Republic of China; 3 MRC Laboratory of Molecular Biology, Cambridge Biomedical Campus, Cambridge, CB2 0QH UK; 4 Department of Biochemistry and Molecular Biology, Third Military Medical University (Army Medical University), 400038 Chongqing, People’s Republic of China

**Keywords:** Infectious diseases, Immunological disorders

Correction to: *Signal Transduction and Targeted Therapy* 10.1038/s41392-020-0148-4, published online 27 March 2020

Since the publication of this article,^1^ we noticed a minor mistake in the reference that needs to be corrected.

To support the clinical manifestation “metabolic acidosis” in the COVID-19, in the fifth sentence of paragraph one, we cited an inappropriate reference 4 “Huang, C. et al. Clinical features of patients infected with 2019 novel coronavirus in Wuhan, China. Lancet. 395, 497-506, (2020)” which needs to be replaced with Diagnosis and Treatment Protocol for COVID-19 (Trial Version 5) published by National Health Commission of the People’s Republic of China.

The key messages of the article are not affected by this correction. We apologize for this inadvertent mistake.
