# Supplementary material for: Lymphopenia predicts disease severity of COVID-19: a descriptive and predictive study
Source: Signal Transduct Target Ther. 2020 Mar 27;5:33. doi: 10.1038/s41392-020-0148-4 (PMC7100419; doi:10.1038/s41392-020-0148-4)
Supplement: Supplementary file 1 — Supplementary information [file 41392_2020_148_MOESM1_ESM.docx]

Supplementary Materials for

Lymphopenia predicts disease severity of COVID-19: a descriptive and predictive study

Li Tan^1,#^, Qi Wang^1,#^, Duanyang Zhang^1^, Jinya Ding^2^, Qianchuan Huang^2^, Yi-Quan Tang^3^, Qiongshu Wang^1*^, Hongming Miao^4*^

Correspondence to: hongmingmiao@sina.com; whzyywqs@163.com

**This PDF file includes:**

Figures. S1 to S2

Table S1 to S3


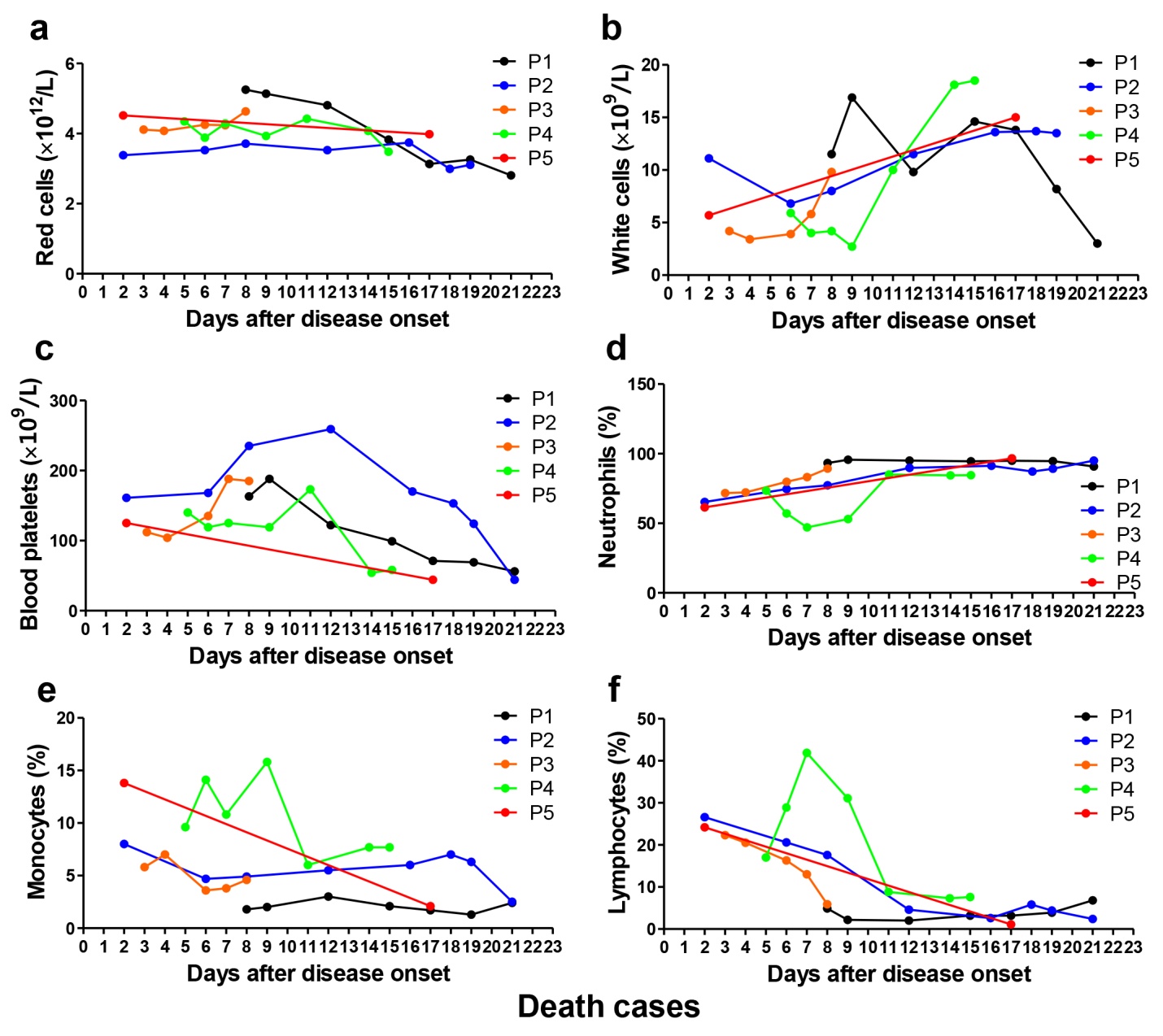


Figure. S1.

S1 Dynamic changes of routine indicators in blood tests of COVID-19 patients

(**a-f**) Dynamic changes of red cells (**a**), white cells (**b**), platelets (**c**), neutrophils (**d**), monocytes (**e**) and lymphocytes (**f**) in the routine blood tests of COVID-19-caused death cases. The descriptive curve of individual patient P1, P2, P3, P4 and P5 was displayed. (n=5)


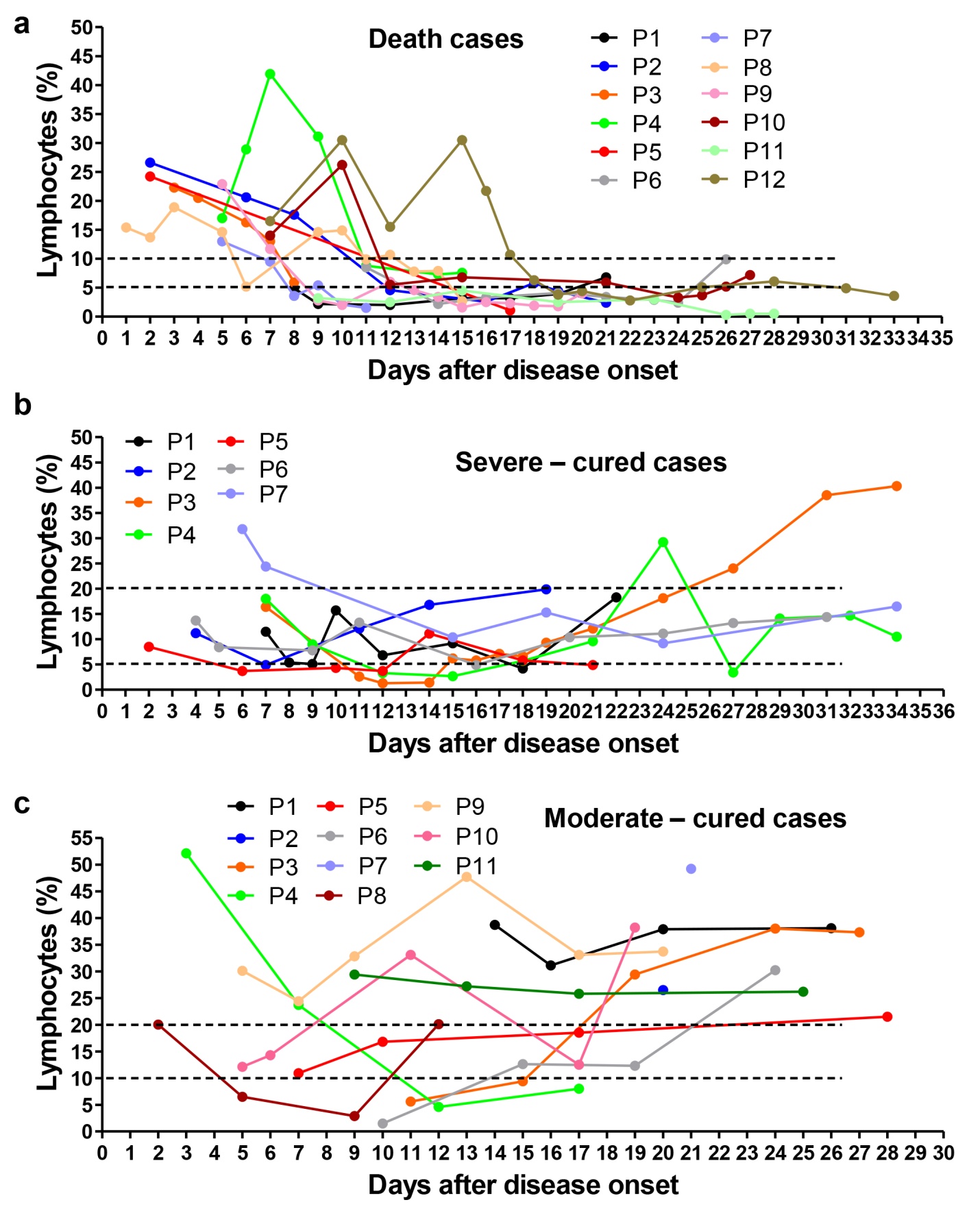


Figure. S2.

Dynamic changes of LYM% in the death, severe-cured and moderate-cured patients with COVID-19

(**a**) Dynamic changes of LYM% in the death cases (n=12). (**b**) Dynamic changes of LYM% in the severe-cured cases (n=7). (**c**) Dynamic changes of LYM% in the moderate-cured cases (n=11). The data of each patient was showed by a descriptive curve. Severe-cured: severe type with a cured outcome; moderate-cured: moderate type with a cured outcome

Table S1.

**Basic information of 12 death cases of COVID-19 patients**

| Code | Age(year) | Gender | Treatment time (day) | Initial symptoms | Maximum temperature (°C) | CT image | Medical history |
| --- | --- | --- | --- | --- | --- | --- | --- |
| P1 | 79 | male | 24 | fever; hypertension | 38.2 | ground-glass shadow of both lungs | hypertension;diabetes ;renal insufficiency |
| P2 | 93 | male | 20 | fever;cough | 40 | ground-glass shadow of both lungs | diabetes;hypertension;coronary heart disease |
| P3 | 96 | male | 10 | diarrhea | 38.9 | Inflammation in both lungs | hypertension;cardiac insufficiency;renal insufficiency |
| P4 | 90 | male | 15 | cough; expectoration | 39 | not detected | hypertension;cardiac insufficiency;renal insufficiency |
| P5 | 96 | male | 17 | fever;fear of cold;cough | 39.3 | ground-glass shadow of both lungs | diabetes;hypertension;coronary heart disease |
| P6 | 31 | female | 25 | muscular soreness | 39.5 | ground-glass shadow of both lungs | healthy |
| P7 | 91 | male | 15 | fever;cough | 38.6 | ground-glass shadow of both lungs | hypertension;coronary heart disease |
| P8 | 76 | male | 15 | fever;muscular soreness | 38.5 | ground-glass shadow of both lungs | liver cirrhosis;osteoporosis; |
| P9 | 40 | male | 20 | fever;cough | 40.2 | ground-glass shadow of both lungs | fatty liver |
| P10 | 73 | male | 26 | fever;cough | 38.7 | Infection of both lungs | fatty liver |
| P11 | 67 | male | 28 | fever;muscular soreness | 38 | inflammation in lower right lung | cholecystectomy;hepatic nodule resection |
| P12 | 84 | male | 28 | fever | 38.2 | ground-glass shadow of both lungs | diabetes;hypertension;coronary heart disease |

n=12; male/female ratio=11:1; average age =76 years; average treatment time: 20 days

Table S2.

**Basic information of 7 cases of severe-cured patients with COVID-19**

| code | Age(year) | Gender | Treatment time(day) | Initial symptoms | Maximum temperature (°C) | CT image | Medical history |
| --- | --- | --- | --- | --- | --- | --- | --- |
| P1 | 77 | female | 37 | fever;cough | 38.2 | ground-glass shadow of both lungs | hypertension |
| P2 | 62 | female | 41 | fever;fear of cold | 40 | ground-glass shadow of both lungs | diabetes;hypertension |
| P3 | 48 | male | 34 | fever;fear of cold | 38.9 | ground-glass shadow of both lungs | hypertension;fatty liver |
| P4 | 56 | male | 37 | fever | 39 | ground-glass shadow of both lungs | tuberculosis |
| P5 | 29 | female | 39 | fever;fear of cold | 39.3 | ground-glass shadow of both lungs | healthy |
| P6 | 31 | male | 30 | fever;cough | 39.5 | ground-glass shadow of both lungs | healthy |
| P7 | 41 | male | 27 | fever;cough | 38.6 | ground-glass shadow of both lungs | healthy |

n=7; male/female ratio=4:3; average age =35 years; average treatment time: 35 days; severe-cured patients: the severe type of patients with a cured outcome

Table S3.

**Basic information of 11 cases of moderate-cured patients with COVID-19**

| Code | Age(year) | Gender | Treatment time (day) | Initial symptoms | Maximum temperature (°C) | CT image | Medical history |
| --- | --- | --- | --- | --- | --- | --- | --- |
| P1 | 40 | female | 24 | fever;cough;fatigue | 37.5 | infections in lower right lungs | healthy |
| P2 | 69 | female | 24 | fever | 38 | ground-glass shadow of both lungs | healthy |
| P3 | 51 | female | 29 | fever | 38.5 | ground-glass shadow of both lungs | hypertension |
| P4 | 31 | male | 26 | muscular soreness | 38.5 | ground-glass shadow of both lungs | healthy |
| P5 | 55 | male | 32 | fever;muscular soreness | 37.7 | ground-glass shadow of both lungs | healthy |
| P6 | 56 | female | 23 | fever;muscular soreness | 38.5 | ground-glass shadow of both lungs | pyelonephritis |
| P7 | 61 | female | 27 | fever;cough;  muscular soreness | 38 | ground-glass shadow of both lungs | healthy |
| P8 | 54 | female | 23 | fever;cough;fatigue | 37.4 | ground-glass shadow of both lungs | healthy |
| P9 | 50 | female | 27 | fever;cough;fatigue | 38 | ground-glass shadow of both lungs | [gynopathy](javascript:;) |
| P10 | 17 | male | 23 | fever;muscular soreness | 38.5 | ground-glass shadow of both lungs | obesity |
| P11 | 53 | female | 28 | cough;  muscular soreness | 37.8 | ground-glass shadow of both lungs | healthy |

n=11; male/female ratio=3:8; average age =49 years; average treatment time: 26 days; moderate-cured patients: the moderate type of patients with a cured outcome
